# Iron Pill Aspiration in the Age of Polypharmacy: Time to Rethink the Risks

**DOI:** 10.7759/cureus.104665

**Published:** 2026-03-04

**Authors:** Felix W Wireko, Prince Ntiamoah

**Affiliations:** 1 Pulmonary and Critical Care Medicine, Mayo Clinic, Rochester, USA; 2 Interventional Pulmonary, Aurora BayCare Medical Center, Green Bay, USA

**Keywords:** airway injury, dysphagia, iron pill aspiration, patient safety, polypharmacy

## Abstract

Iron pill aspiration represents an underrecognized but clinically significant cause of airway injury, particularly in the context of aging populations and increasing polypharmacy. While foreign body aspiration is commonly associated with food particles and small objects, medicinal pills such as ferrous sulfate present a distinct risk due to their corrosive biochemical properties. Oxidative injury resulting from ferrous ion conversion can cause mucosal necrosis, airway inflammation, and long-term complications, including bronchial stenosis and lobar collapse. Diagnosis is often delayed because aspiration events are typically unwitnessed, initial symptoms are nonspecific, and standard imaging modalities lack sensitivity.

This editorial reframes iron pill aspiration as a preventable adverse drug event and explores its association with presbyphagia, medication burden, and patient safety. It summarizes current evidence on pathophysiology, diagnostic challenges, and clinical outcomes, with an emphasis on the need for proactive prevention. Recommended strategies include deprescribing medications that impair swallowing, reducing pill burden through alternate-day dosing, and incorporating dysphagia screening into routine prescribing practices. For patients at higher risk, considering liquid preparations may also help reduce harm.

As iron supplementation remains central to anemia management worldwide, clinicians must balance therapeutic benefit with airway safety. Iron pill aspiration should no longer be considered rare but a foreseeable complication requiring systematic prevention efforts globally.

## Editorial

Introduction

In the silent intersection of aging, polypharmacy, and overlooked dysphagia lies a hidden but preventable airway crisis.

Foreign body aspiration is frequently associated with objects such as peanuts, coins, or dental appliances. However, iron pill aspiration, though less conspicuous, represents a significant and often overlooked clinical concern. With increasing global medication use and an aging population, pill aspiration is becoming more prevalent. It constitutes an underrecognized indicator of iatrogenic harm that necessitates heightened diagnostic awareness and systemic intervention [1].

While food and small objects remain the most commonly aspirated materials, medicinal pills are increasingly identified as significant contributors to airway compromise in adults. Medications account for up to 7% of foreign body aspirations [2]. In the largest published case series, 11 cases of iron pill aspiration were documented over a 10-year period, with a disproportionate incidence among elderly patients [3]. This paucity of reported cases likely reflects significant underdiagnosis rather than true rarity.

Between 1999 and 2012, prescription medication use among US adults increased from 51% to 59%, and the prevalence of polypharmacy (defined as the use of five or more drugs) nearly doubled from 8.2% to 15% [1]. Polypharmacy increases the risk of aspiration and is independently associated with malnutrition and nutritional deficiencies in elderly populations. Among institutionalized older adults, nearly 25% receive 10 or more medications daily [4].

Oropharyngeal dysphagia, affecting nearly 30% of older adults, is a major risk factor for aspiration. However, individuals without dysphagia or neurological disease also remain at risk. Studies indicate that 50% of community-dwelling adults aged 70 years or older exhibit presbyphagia, an age-related decline in swallowing coordination, even in the absence of overt disease [5].

Oral iron supplementation, particularly ferrous sulfate, is commonly prescribed for iron-deficiency anemia due to its affordability and accessibility [6]. However, ferrous sulfate poses significant risks. Unlike inert aspirated objects, ferrous sulfate tablets cause rapid, corrosive injury upon contact with the airway mucosa. The oxidation of ferrous (Fe²⁺) to ferric (Fe³⁺) iron promotes the formation of reactive oxygen and nitrogen species, leading to local oxidative stress and tissue injury, which may manifest as mucosal inflammation, necrosis, and ulceration [7].

In a recent series by Xing et al. [3], all patients who aspirated iron tablets required bronchoscopic or surgical intervention, some of whom had irreversible sequelae, including bronchial stenosis, lobar collapse, or vocal cord damage. The injuries from ferrous sulfate are not only severe but distinct from those caused by other pills like potassium chloride or bisphosphonates. The depth and rapidity of necrosis, sometimes within hours, call for urgent recognition and removal. In one reported case, a tablet lodged in the larynx for only a few hours led to voice loss and required vocal fold augmentation [8].

Diagnostic challenge

Diagnosis is challenging. Patients seldom recall aspiration events, and initial symptoms often resemble those of common pulmonary conditions. Standard imaging frequently fails to provide definitive findings. Chest radiography identifies only 41% of foreign body aspirations [9]. Although chest CT is highly sensitive for conventional foreign bodies, its utility in iron pill aspiration is limited. Iron tablets dissolve and fragment rapidly in the airway, typically leaving no distinct radiopaque material, which renders direct visualization on CT unreliable. In the 11-case series by Xing et al., chest CT findings were mostly nonspecific and primarily revealed secondary obstructive changes, such as bronchial wall thickening, bronchial stenosis or occlusion, and consolidation, rather than direct evidence of aspirated material. Bronchoscopy remains the gold standard for early retrieval and assessment of injury, but it is often performed too late. In cases of delayed diagnosis, biopsies may reveal iron deposits in damaged mucosa, providing a frequently overlooked forensic clue in otherwise unexplained respiratory deterioration.

Prevention and safer practice

Given the irreversible complications of IPA in an era focused on medication safety, deprescribing, and person-centered care, iron pill aspiration should not be a forgotten footnote. Prevention is both feasible and urgent. Strategies include deprescribing medications that impair swallowing, such as anticholinergics, sedatives, and antipsychotics; substituting safer formulations, including liquid iron or dispersible tablets, for patients with dysphagia or elevated aspiration risk; and adopting alternate-day iron dosing, which can improve tolerability and reduce pill burden without compromising efficacy.

Slower iron dissociation appears to reduce free radical generation and oxidative injury. Consequently, slow-release formulations such as liposomal iron may limit the rapid release of free Fe²⁺ ions responsible for mucosal oxidative damage and could represent an alternative to ferrous sulfate [10]. However, further research is necessary to determine their safety in cases of aspiration. 

Managing older adults on iron supplements requires coordinated input from pharmacists, speech-language pathologists, nurses, and physicians to screen for dysphagia, adjust medications, and educate on the risk of aspiration.

Iron pill aspiration: challenges, opportunities, and future directions

Iron pill aspiration is associated with severe airway injury and significant long-term consequences. Case reports and small series demonstrate a disproportionate impact on older adults experiencing polypharmacy. Despite these findings, research remains limited, fragmented, and frequently retrospective or underpowered. Most cases are recognized only after serious complications arise, leaving substantial knowledge gaps regarding the true incidence, risk stratification, and effective prevention strategies.

Ongoing research aims to reframe iron pill aspiration as a recognized and preventable cause of catastrophic airway injury, rather than a rare clinical curiosity. The primary objective is to integrate aspiration risk into prescribing decisions, especially for elderly patients, by prioritizing safer iron formulations and reducing polypharmacy burden.

Prospective studies are necessary to determine prevalence, validate risk factors, and compare outcomes among various iron formulations. Translational research into the biochemical mechanisms of mucosal injury may inform the development of safer drugs. The primary challenge is clinical invisibility: aspiration events are rarely observed, initial symptoms are nonspecific, and standard imaging often fails to detect them. Addressing this issue will require enhanced dysphagia screening, multidisciplinary care pathways, and increased clinician awareness.

Formulation-based prevention represents a promising area of focus. Replacing ferrous sulfate tablets with slow-release or liquid formulations may potentially reduce the risk of iron pill aspiration while maintaining therapeutic efficacy. Incorporating this approach into polypharmacy management strategies could significantly enhance patient safety.

Conclusion

Although the mucosal toxicity of iron is well established in gastroenterology, its impact on the airway remains underrecognized. In light of an aging population and increasing polypharmacy, iron pill aspiration should be regarded not as a rare occurrence but as a preventable cause of severe airway injury that requires multidisciplinary attention and the implementation of proactive, system-wide strategies to improve prescribing safety. Clinicians should integrate aspiration risk into prescribing decisions, particularly for older adults, by choosing safer formulations, reducing pill burden, and routinely assessing swallowing function. We urge clinicians, policymakers, and educators to collaborate in preventing this avoidable and potentially catastrophic complication.
